# Psychological Pathways Linking Parent-Child Relationships to Subjective and Objective Sleep Among Older Adults

**DOI:** 10.1093/geroni/igaa057.1926

**Published:** 2020-12-16

**Authors:** Haowei Wang, Kyungmin Kim, Jeffrey Burr, Bei Wu

**Affiliations:** 1 University of Massachusetts Boston, Boston, Massachusetts, United States; 2 New York University, New York, New York, United States

## Abstract

This study investigated whether older adults’ relationships with their children were associated with their self-reported subjective sleep quality and actigraphy-measured objective sleep characteristics, as well as whether depressive symptoms and loneliness mediated the association between these parent-child relationships and sleep. Data were taken from the second wave of the National Social life, Health, and Aging Project, in which 569 respondents (age 57-85) participated in the sleep module, along with the social network module that provided relationship information for participants and their children. Results from structural equation modeling showed that (1) parents’ closeness with children was associated with better objective sleep (i.e., fragmentation of sleep and percent sleep), (2) more frequent contact with children was related to better subjective sleep quality, (3) depressive symptoms and loneliness were associated with worse subjective sleep quality, and (4) less closeness with children were related to worse subjective sleep quality via older adults’ depressive symptoms. Part of a symposium sponsored by the Sleep, Circadian Rhythms and Aging Interest Group.

